# Genomic structure and marker-derived gene networks for growth and meat quality traits of Brazilian Nelore beef cattle

**DOI:** 10.1186/s12864-016-2535-3

**Published:** 2016-03-15

**Authors:** Maurício A. Mudadu, Laercio R. Porto-Neto, Fabiana B. Mokry, Polyana C. Tizioto, Priscila S. N. Oliveira, Rymer R. Tullio, Renata T. Nassu, Simone C. M. Niciura, Patrícia Tholon, Maurício M. Alencar, Roberto H. Higa, Antônio N. Rosa, Gélson L. D. Feijó, André L. J. Ferraz, Luiz O. C. Silva, Sérgio R. Medeiros, Dante P. Lanna, Michele L. Nascimento, Amália S. Chaves, Andrea R. D. L. Souza, Irineu U. Packer, Roberto A. A. Torres, Fabiane Siqueira, Gerson B. Mourão, Luiz L. Coutinho, Antonio Reverter, Luciana C. A. Regitano

**Affiliations:** Embrapa Agricultural Informatics, Av. André Tosello, 209, Campinas, SP Brazil; Embrapa Southeast Livestock, Rodovia Washington Luiz, Km 234, São Carlos, SP Brazil; Commonwealth Scientific and Industrial Research Organization - Agriculture, 306 Carmody Road, Brisbane, QLD Australia; Department of Genetics and Evolution, Federal University of São Carlos, Rodovia Washington Luiz, Km 235, São Carlos, SP Brazil; Embrapa Beef Cattle, Av. Rádio Maia, 830, Campo Grande, MS Brazil; State University of Mato Grosso do Sul, Rodovia Uems-Aquidauana km 12, Aquidauana, MS Brazil; Department of Animal Science, University of São Paulo, Av. Padua Dias, 11306 Piracicaba, SP Brazil; Faculdade de Medicina Veterinaria e Zootecnia, Federal University of Mato Grosso do Sul, Av. Senador Filinto Müller, 2443 Campo Grande, MS Brazil

**Keywords:** Genotyping, AWM, PCIT, GWAS

## Abstract

**Background:**

Nelore is the major beef cattle breed in Brazil with more than 130 million heads. Genome-wide association studies (GWAS) are often used to associate markers and genomic regions to growth and meat quality traits that can be used to assist selection programs. An alternative methodology to traditional GWAS that involves the construction of gene network interactions, derived from results of several GWAS is the AWM (Association Weight Matrices)/PCIT (Partial Correlation and Information Theory). With the aim of evaluating the genetic architecture of Brazilian Nelore cattle, we used high-density SNP genotyping data (~770,000 SNP) from 780 Nelore animals comprising 34 half-sibling families derived from highly disseminated and unrelated sires from across Brazil. The AWM/PCIT methodology was employed to evaluate the genes that participate in a series of eight phenotypes related to growth and meat quality obtained from this Nelore sample.

**Results:**

Our results indicate a lack of structuring between the individuals studied since principal component analyses were not able to differentiate families by its sires or by its ancestral lineages. The application of the AWM/PCIT methodology revealed a trio of transcription factors (comprising VDR, LHX9 and ZEB1) which in combination connected 66 genes through 359 edges and whose biological functions were inspected, some revealing to participate in biological growth processes in literature searches.

**Conclusions:**

The diversity of the Nelore sample studied is not high enough to differentiate among families neither by sires nor by using the available ancestral lineage information. The gene networks constructed from the AWM/PCIT methodology were a useful alternative in characterizing genes and gene networks that were allegedly influential in growth and meat quality traits in Nelore cattle.

**Electronic supplementary material:**

The online version of this article (doi:10.1186/s12864-016-2535-3) contains supplementary material, which is available to authorized users.

## Background

Brazilian livestock began with taurine cattle introduced from Europe in the colonial period, around 450 years ago. Several years later, starting in 1930 but mainly in 1960 and 1962, a *Bos indicus* cattle breed named Nelore was introduced in Brazil from India and the herd expanded to the majority of the territory due to its good adaptability to tropical climate. It is estimated that 7000 Nelore cattle were introduced from India, the majority were descendants from 6 main sires named Karvadi, Taj Mahal, Kurupathy, Golias, Godhavari and Rasta [1]. The Nelore, a breed with white or gray pelage and short horns, is nowadays the most representative breed in Brazil and dominates the Brazilian beef cattle production with more than 75 % of the total herd which equates to more than 130 million heads of Nelore cattle. Artificial insemination is widely used in Brazil [2] and semen for only a few sires has been intensively used nationwide. With the advent of the Illumina BovineHD BeadChip (Illumina Inc., San Diego, CA), a high density SNP panel comprising over 770,000 markers, we were able to measure the genomic diversity of the Brazilian Nelore from the main commercial sire families and characterize their genomic relationship and genetic profiles across a series of phenotypes. Traditional phenotype-based selection has been focused primarily on improving growth and maternal traits [3]. However, marker-assisted or genomic selection is particularly valuable for traits that have late, onerous and expensive measurement such as meat quality, yield and feed efficiency [4]. Despite production advantages of *Bos indicus* composite breeds in tropical and subtropical environments, the inferiority of beef quality, especially tenderness, is a worrying issue for producers [5, 6]. It is known that Nelore breed show moderate heritability for meat quality [7] as well as for growth-related traits [8], which make the application of genomic tools feasible.

Genome-Wide Association Studies, or GWAS, are today a common practice in order to find associations of genome regions to phenotypes of interest such as meat quality traits [7, 9, 10]. However, GWAS are sometimes elusive regarding the low power of association of the markers to the trait and also to the association of regions of the genome that lack apparent relation to the trait being studied [11, 12]. Some meta-analysis approaches are appealing as they attempt to enrich GWAS analyses with information from other sources. One of these methods is the system’s biology AWM/PCIT methodology, which involves the construction of SNP-based gene network interactions, integrating results from several GWAS by means of the Association Weight Matrices (AWM) and Partial Correlation and Information Theory (PCIT) [13–16]. We took advantage of this methodology to explore growth and meat quality traits from 780 samples of Brazilian Nelore cattle, systematically sourced to represent 34 highly influential sires and their 746 half-sibs from across Brazil. Further, we inferred a gene network focused on growth and meat quality traits and found several genes implicated in the regulation of these phenotypes.

## Results

### Genetic profile of Brazilian Nelore beef cattle

**Fig. 1:**
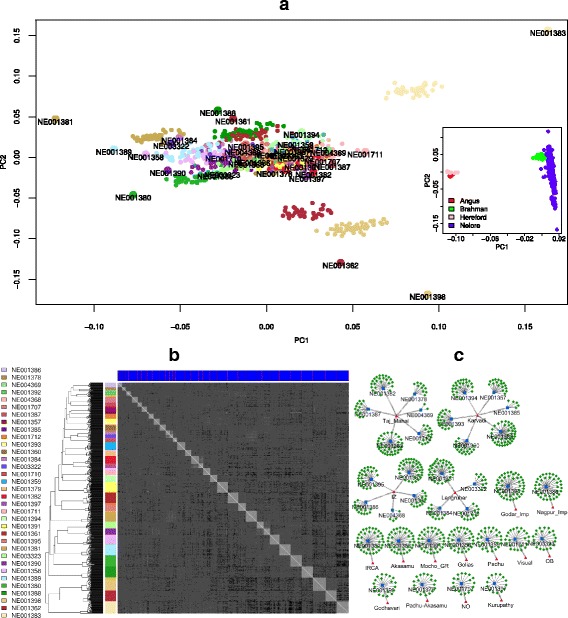
Genetic profiling using the Genomic Relationship Matrix. **a** PCA analysis of all 780 Nelore (families differentiated by colors, sires are labeled). Inset shows a PCA with other breeds from Hapmap. **b** Heatmap and hierarchical clustering of the 780 Nelore. Lateral palette colors represent the families (same color correspondence to (**a**); upper color palette differentiates sires (*red*) from sibs (*blue*); shades of grey from the heatmap represent relationship similarities (darker is less related). **c** Pedigree view of the families showing the sires (*blue*), sibs (*green*) and the lineage ancestral from father side (*red*)

**Fig. 2:**
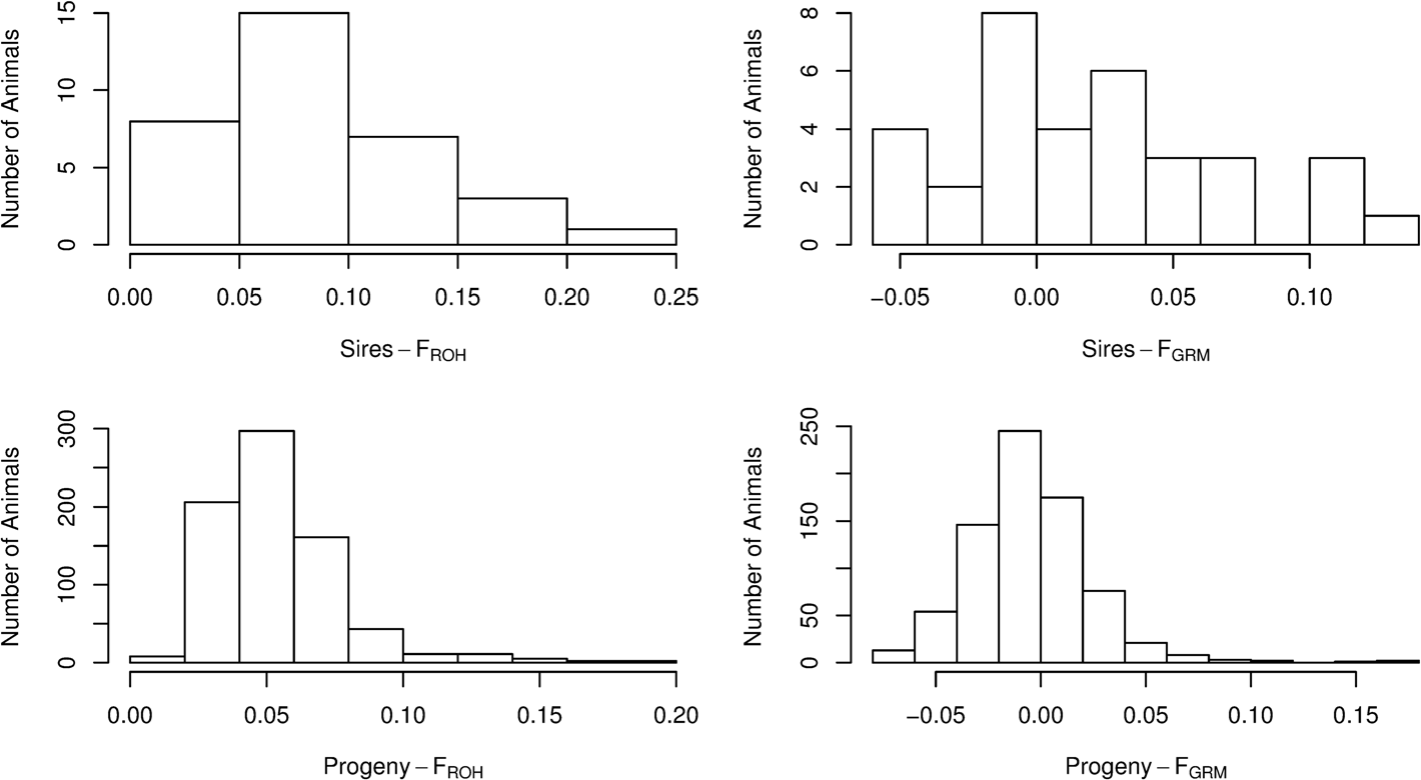
Genomic inbreeding coefficients. Runs of homozygosity (FROH) estimations with a minimum length of 30 SNPs, and inbreeding coefficient derived from a genomic relationship matrix (FGRM) for sires and its progeny

After quality control filters we retained genotypes of 449,203 SNPs for linkage disequilibrium and haplotype block analysis, and 224,969 TagSNPs from 780 Nelore animals, including 34 prominent sires and their 746 progeny (Table 1). These genotypes were coupled with genotypes of the same SNP for a sample of 46 Brahman, Hereford and 44 Angus cattle sourced from the Bovine HapMap project [17, 18], and used to calculate a genomic relationship matrix (GRM) that was used in a principal component analysis (PCA) [19]. This analysis (Fig. 1a, inset) shows that the first component, explaining 86 % of the variation in genotype profiles, separates the taurine (Angus and Hereford) from the indicine (Brahman and Nelore) breeds, indicating that the genotyping data is reliable. A GRM considering only the 780 Nelore animals and the 224,969 TagSNPs was also constructed and used for a PCA analysis exclusive for the Brazilian Nelore sample. The results for the PCA (Fig. 1a) shows that although some half-sib families are well distinguished (e.g. the NE001383, NE001398 and NE001362 families), the bulk of the samples were not well separated by the first component, indicating a lack of structuring. The first and second components only explain 8.1 % and 6.8 % of the variation respectively. It is interesting to note in Fig. 1a that the distance from a given sire (greater labeled circles) to its corresponding cluster of half-sibs is visually symmetrical to the distance of the half-sib cluster to the center of the figure. We suggest that this effect is due to the randomness diversity of the genetic effect of the dams that would bring more related individuals to the center of the figure, indicating that these sires are less related to its half-sib families and even less related to the bulk of more related individuals in the center. The average genomic inbreeding coefficient from the GRM (FGRM) reports values of 2 % (± 5 % SD) for sires and 0 % (± 3 % SD) for progeny. Also, inbreeding coefficient derived from runs of homozygosity (FROH) reports values of 9 % (± 5 % SD) for sires, and 6 % (± 2 % SD) for progeny, showing that the 34 sires are more inbred than their progeny, and that the random genetic pool from the dams brought more variability into this sample. The distribution of these inbreeding coefficients (FGRM and FROH) can be seen in Fig. 2. Additional file 1: Figure S1C depicts the pedigree with the information of the dams showing that some dams participate in more than one family.

**Table 1:** Genetic background of the sires

Sire ID	Number of Sibs	mtDNA	Lineage	Lineage descendants (Sire + Sibs)
NE001388	36	Taurus	Akasamu	37
NE001398	43	Indicus	Godar Imp.	44
NE001390	20	Taurus	Godhavari	21
NE001358	31	Taurus	Golias	32
NE001389	36	Taurus	IRCA	37
NE001361	35	Indicus	IZ	96
NE001386	14	Taurus	IZ	
NE001392	13	Taurus	IZ	
NE001395	25	Indicus	IZ	
NE004368	4	–	IZ	
NE001357	17	Taurus	Karvadi	129
NE001360	19	Taurus	Karvadi	
NE001383	40	Taurus	Karvadi	
NE001385	5	Taurus	Karvadi	
NE001393	19	Taurus	Karvadi	
NE001394	23	Taurus	Karvadi	
NE001397	14	Indicus	Kurupathy	15
NE001381	36	Taurus	Lengruber	76
NE001384	12	Taurus	Lengruber	
NE001710	14	Taurus	Lengruber	
NE003322	10	–	Lengruber	
NE001391	34	Taurus	Mocho GR	35
NE001380	37	Taurus	Nagpur Imp	38
NE001707	18	Indicus	NO	19
NE003323	22	–	OB	23
NE001359	26	Taurus	Padhu	27
NE001379	19	Taurus	Padhu-Akasamu	20
NE001362	36	Indicus	Taj Mahal	107
NE001378	10	Taurus	Taj Mahal	
NE001382	24	Taurus	Taj Mahal	
NE001387	13	Taurus	Taj Mahal	
NE001712	16	Indicus	Taj Mahal	
NE004369	2	–	Taj Mahal	
NE001711	23	Taurus	Visual	24
TOTAL	746	7 Indicus (23.33 %)	17	780
		23 Taurus (76.66 %)

In addition to the PCA, we performed a hierarchical cluster analysis of the GRM and plotted the resulting heatmap (Fig. 1b). This analysis shows that the 34 families are well distinguished in the heatmap (grey squares in the central diagonal) and the sire relatedness to its sibs can be distinguished in some cases by a white cross inside the central squares (e.g. NE001710 family) or a white border line for some squares that follows the red lines in the upper palette representing the sires. In overall the heatmap is greyish than what would be expected taking into account that the sires were selected to be more unrelated with each other, suggesting that the half-sib families are genetically related. Wright’s F statistic (Fst) was applied to evaluate the degree of genetic differentiation among each sire with its progeny as one population (34 populations) and each lineage with its descendants as one population (17 populations) yielding values of Fst = 0.14 and Fst = 0.09, respectively, which can be considered a small level of genetic differentiation in this Nelore sample [20]. The clustering analysis also put together the majority of the animals evidencing the relatedness of the families. Some darker grey shades can be seen within the progeny of individual NE001383, which is the individual that is more distant in the PCA analysis, showing that this sire and its half-sibs are more unrelated to all other individuals. Although this individual sire is from the Karvadi lineage and supposed to be related to the NE001394, NE001393, NE001360 and NE001385 (see Fig. 1c), in the PCA it is more distinguished from others. Overall, the lineage information obtained from pedigree was not useful to evidence any kind of relatedness between individuals with similar ancestry. For example, nor the PCA or the hierarchical clustering show any improvement in distinguishing related and unrelated animals regarding to its lineages (Additional file 1: Figure S1A and S1B). The hierarchical clustering also does not cluster individuals according to its ancestral lineages (see for example the cluster of individuals NE001383, NE001362 and NE001398, from the Karvadi, Taj Mahal and Godar Imp lineages). One explanation for the failure of these analyses to differentiate the half-sib families is the fact that the families do not have the same number of animals and the same goes for the lineage ancestry of the families (see Table 1). These distributions could affect the effectiveness of the GRM to provide enough information to generate a good differentiation in the PCA or hierarchical cluster analyses across lineages.

We obtained information of mitochondrial DNA (mtDNA) of 30 of the 34 sires from a previous study [21]. Results show that 23 (76.66 %) of these have *Bos taurus* maternal ancestry (Table 1), which agrees with the hypothesis that most of the Brazilian Nelore herd was obtained by backcrossing with females of taurine origin [22]. Although two of the most divergent sires, NE001398 and NE001362 were of indicine maternal ancestry, in overall the maternal ancestry seems not to have influenced the PCA results. For a better characterization of the genomic data under study we analyzed the haplotype structure of the population and estimated the extent of the linkage disequilibrium (LD), decay by physical genomic distance on autosomes, as the squared correlation coefficient (*r*^2^) between SNP pairs. Additional file 2: Figure S2 shows that LD varies from *r*^2^ = 0.58 between SNPs distance up to 1 kb for BTA5 to *r*^2^ = 0.03 between SNPs distance of 500 kb to 1 Mb for BTA25. The average distance between SNPs for this LD study was 5.62 kb, with large gaps of 1.08 Mb on BTA7, and of 0.9 Mb and 0.84 Mb on BTA12 and on BTA27, respectively. At the distance of 10 kb to 25 kb, the overall average *r*^2^ was ≈ 0.30, which is considered strong LD and can be used for QTL mapping [23], while at distance of 25 kb to 50 kb, the overall average *r*^2^ was ≈ 0.20, which is sufficient to provide an accuracy of 0.85 for estimation of genomic breeding value [24]. A summary of the haplotype block description is provided on Additional file 3: Table S1. From the SNPs considered for the LD and haplotype block study, 335,179 (75 %) were clustered into haplotype blocks constituted of 3 or more SNPs, spanning 1.35 MB (54 %) of the total autosomal genome size. There were 54,461 haplotype blocks with overall average length of 24.25 kb, BTA16 (882 kb) followed by BTA4 (783 kb), and BTA5 (669 kb) presented the longest haplotype blocks, the average number of SNPs into haplotype blocks was 6, with a maximum of 111 SNPs (BTA18). The haplotype block size and distribution along autosomes is provided in Additional file 4: Figure S3.

The effective population size (Ne) was estimated to be around 214 animals in average per chromosome, with a confidence interval of 13.5, one generation ago (assuming ≈ 7 years for each generation). This number is higher than the Ne obtained from pedigree records, varying from 98 to 68, calculated from past periods of time (from 1979 to 1998) [25] and from another study also using pedigree records, that shows Ne to be around 120 for a Brazilian Polled Nelore breed [26]. We estimated Ne to be higher 10 generations ago (around 270 ± 16.86 animals, in average per chromosome), when the breed was introduced in Brazil. Ne shows a decreasing pattern over the last generations especially for the last two (see Additional file 5: Figure S4).

### Gene networks for growth and meat quality traits

#### Data inspection

We performed correlation analyses using the phenotype collection (see Table 2 for summary statistics) with the VCE software [27]. Although we obtained low heritability values (h^2^ < 0.10) for lightness of meat (LM), lightness of fat (LF), pH (PH), cooking loss (CLO) and dressing (DRE), higher estimates were observed for rib eye area (REA; h^2^ = 0.55), total carcass weight (TCW; h^2^ = 0.26) and back fat thickness (BFT; h^2^ = 0.15). There was a negative correlation between BFT and REA (−0.735) and between BFT and TCW (−0.774) and a strong positive correlation between TCW and REA (0.774) which makes biological sense indicating that the data is consistent. We calculated the influence (1 % increase) of inbreeding over the phenotypes, by estimating the inbreeding depression coefficients derived from FROH and FGRM (Table 3). Only BFT, PH and CLO appeared to be significantly affected by inbreeding although this was not a consensus among the two methods of estimation. The results suggest that inbreeding did not strongly influentiate the traits.

**Table 2:** Trait data and GWAS results information

Trait	*n*	min	max	avg	SD	h^2^	*p* ≤ 10^−3^	*p* ≤ 10^−4^	*p* ≤ 10^−5^
TCW	671	182.5	346.8	250.26	27.88	0.262	1246	219	32
						(0.114)	(17.97)	(10.26)	(7.03)
DRE	671	42.6	86.5	56.22	3.63	0.092	680	100	20
						(0.080)	(33.02)	(22.49)	(11.25)
REA	669	39.24	84.43	60.45	7.26	0.557	826	157	31
						(0.130)	(27.16)	(14.32)	(7.26)
BFT	669	0.07	20	6.16	2.25	0.154	1723	305	56
						(0.112)	(12.97)	(7.37)	(4.02)
LM	671	33.5	49.43	40.09	3.18	0.037	957	218	64
						(0.050)	(23.43)	(10.31)	(3.51)
LF	671	12.02	40.21	19.94	5.41	0.097	1388	340	96
						(0.663)	(16.12)	(6.61)	(2.34)
PH	671	9.48	24.28	15.31	3.15	0.040	928	215	56
						(0.067)	(24.17)	(10.45)	(4.02)
CLO	671	16.54	84.36	75.65	4.45	0.041	939	138	22
						(0.057)	(23.88)	(16.29)	(10.22)

*TCW, Kg* Total Carcass Weight, *DRE, %* Dressing, *REA, cm* ^*2*^ Rib Eye Area, *BFT, mm* Back Fat Thickness, *LM* Lightness of Meat, *LF* Lightness of Fat, *PH* pH, *CLO, %* Cooking Loss. n is the number of animals used, min. is the minimum value, max is the maximum value, avg is the average value, SD is the standard deviation, h^2^ is the heritability (standard error inside brackets), *p* ≤ 10^−3^ is the number of SNPs under this *p*-value, *p* ≤ 10^−4^ is the number of SNPs under this *p*-value, *p* ≤ 10^−5^ is the number of SNPs under this *p*-value. Inside parentheses is the FDR threshold (%) for each given significance [78]

**Table 3:** Estimates of inbreeding depression for meat quality traits. Estimates expressed as change in adjusted phenotype per 1 % increase, using a inbreeding coefficient derived from a genomic relationship matrix (FGRM), and a inbreeding coefficient derived from runs of homozygosity (FROH)

			FGRM		FROH	
Trait	Mean^a^	SD	Estimate	SE	Estimate	SE
LM	−0.249	3.90	4.42	5.11	8.98	6.33
LF	−0.446	7.19	10.39	9.41	9.02	11.68
PH	−0.073	0.66	2.00**	0.86	0.91	1.07
CLO	−0.357	5.30	11.66*	6.93	−5.14	8.61
DRE	−0.336	5.63	10.78	7.37	9.72	9.14
REA	−0.541	8.71	16.88	11.39	−1.37	14.15
BFT	−0.053	2.08	−3.82	2.71	−7.79**	3.36
TCW	−1.467	30.36	6.42	39.76	26.34	49.30

*P < 0.1; **P < 0.05; ^a^adjusted phenotype; *SD* standard deviation, *SE* standard error, *LM* Lightness of Meat, *LF* Lightness of Fat, *PH* pH, *CLO* Cooking Loss, *DRE* Dressing, *REA* Rib Eye Area, *TCW* Total Carcass Weight, *BFT* Back Fat Thickness

#### Genome-wide association studies

**Fig. 3:**
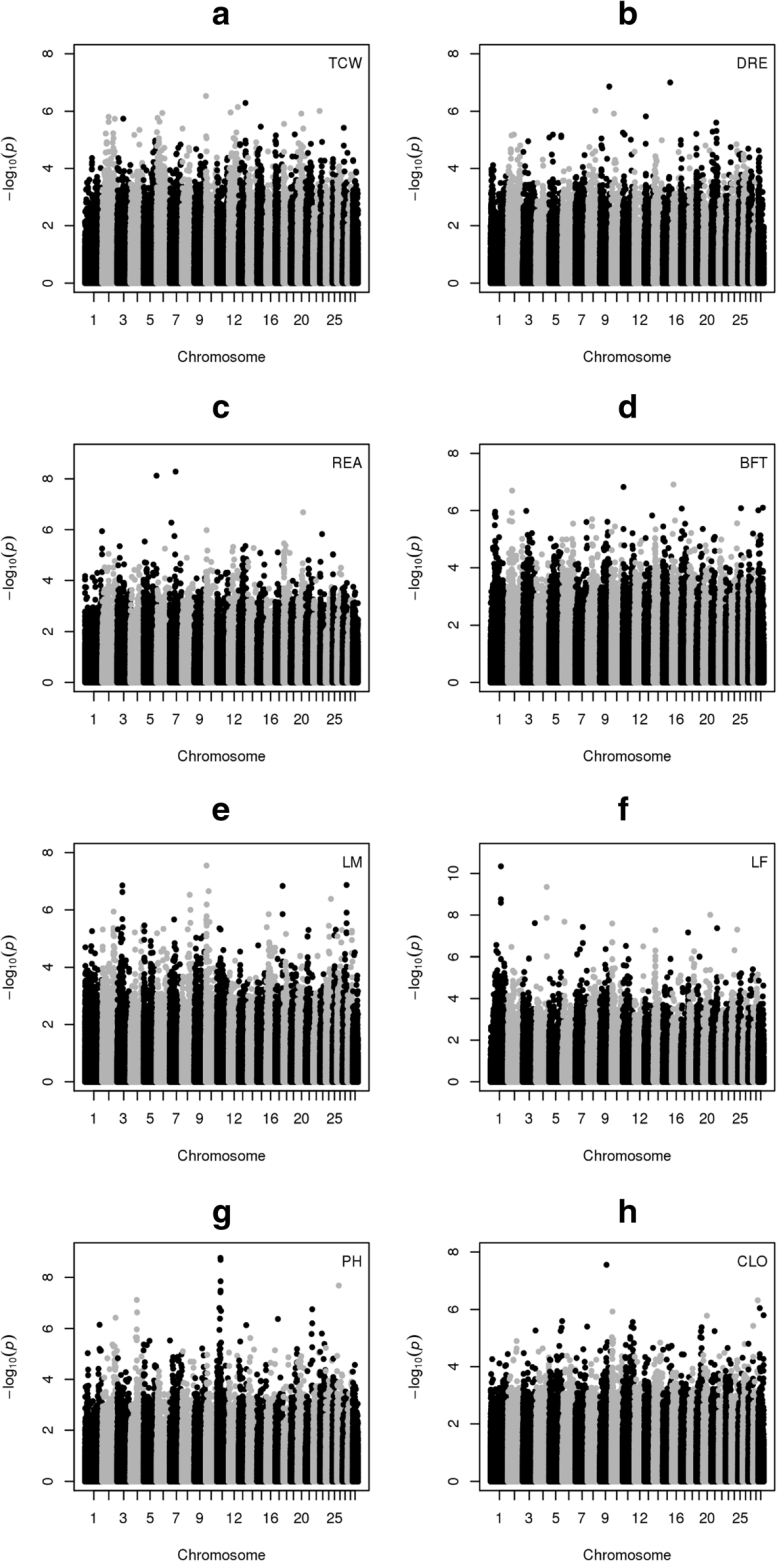
GWAS results for the eight traits. **a**-**h** panels show the manhattan plots for: **a** Total Carcass Weight (TCW); **b** Dressing % (DRE); **c** Rib Eye Area (REA); **d** Back Fat Thickness (BFT); **e** Lightness of Meat (LM); **f** Lightness of Fat (LF); **g** pH (PH); **h** Cooking Loss (CLO)

The QXPAK.5 software was used [28] to perform genome wide association studies (GWAS) using the 224,969 TagSNPs for all eight phenotypes. Manhattan plots with the results are displayed in Fig. 3. When looking for markers with a significance threshold of *p* ≤ 10^−3^ we obtained several hundreds of significant TagSNPs, varying from 1723 (BFT) to 680 (DRE). This high number of significant markers using this significance threshold indicates some inflation of the *p*-values (see Additional file 6: Figure S5 for QQ-plots). If the threshold is pushed further to *p* ≤ 10^−4^ the number of significant TagSNPs fall to a few hundreds and still if a more stringent threshold is used (*p* ≤ 10^−5^) we obtain just several dozens of significant TagSNPs (see Table 2). Although we had some dozens of SNPs with very low p-values (below 10^−5^), the traits that had better heritabilities, like BFT, REA and TCW showed a scattered distribution of significant SNPs over the chromosomes. Other traits showed more specific regions with significant SNPs like the pH trait, showing chromosome 11 with a visual peak of significant SNPs (markers BovineHD1100009381, BovineHD1100009391, BovineHD1100009772, Hapmap25798-BTA-126388, BovineHD1100009860, p-values lower than 10^−8^) in the region of 31 to 32 Mb, pointing to the Follicle Stimulating Hormone Receptor (FSHR) gene, but no evidence was found in the literature linking this gene to pH of meat. Other traits like LM have some diffuse peaks in chromosomes 3, 8, 10 and 27, and LF with some less intense peaks in chromosomes 1 and 14, but the lower heritability of these traits discouraged more profound analyses of these regions.

We did a literature survey of the top 10 genes associated to the lowest p-value TagSNPs for BFT, REA and TCW. For the later we searched for the involvement of the significant genes that could influence the total carcass weight phenotype. We found the gene Serotonin Receptor 2, (HTR2A) associated to the marker BovineHD1200005266 (BTA12, *p* = 1e-6) which plays a role in appetite control [29] and was found associated to birth weight in humans [13]. We also found the Leukemia inhibitory factor receptor (LIFR) gene, associated to the marker BovineHD2000010215 (BTA20, *p* = 1.21e-6) which is involved in skeletal growth and bone formation and resorption in humans [30]. For the BFT phenotype we searched for genes involved in studies related to adipose tissue and fat. We found the gene EPHA6 (ephrin receptor a6), associated to the marker BovineHD0100011825 (BTA1, *p* = 1.10e-6) found associated in a copy number variation (CNV) study to childhood obesity [31] and the gene TMEM182 (transmembrane protein 182), associated to the marker BovineHD1100002798 (BTA11, *p* = 1.5e-7) which is found up-regulated in brown adipose tissue during adipogenesis and myogenesis [32] and also found associated in another GWAS for BFT [7], discussed below. We found no specific genes related to REA in the literature, that could be influencing muscle growth and myogenesis, associated to any TagSNPs of the top 10 list. In a previous work from our group [7], GWAS were performed using a Bayesian approach, with a subset of the data used in this work (smaller number of animals and traits). We compared the set of SNP, associated with significance below 10^−5^ in our work, against the ones associated in this previous work, to try to find genes simultaneously associated in both studies. We found one common gene for LM, 37 common genes for LF, 15 common genes for BFT and 12 genes for REA (gene ids at Additional file 7: Spreadsheet S1). The accordance between both works for BFT, REA and LF were of 26 %, 38 % and 38 % respectively, of all genes with significance below 10^−5^. We could not estimate the accordance for the TCW trait as it was not used in this previous work.

To test for Gene Ontology (GO) enrichment, we ordered all TagSNPs according to the pleiotropy test [33] used. The ordered list of all corresponding genes was used as input to GOrilla [34] and results showed “cell adhesion” (GO:0007155, false discovery rate or FDR q-value = 2.8e-4), “biological adhesion” (GO:0022610, FDR q-value = 1.52e-4) and “developmental growth” (GO:0048589, FDR q-value = 1.29e-4) as the three GO terms from the biological process tree enriched with significant values after a FDR correction. Cell adhesion was also found significantly enriched for color of muscle and fat and for meat tenderness in our previous work [7]. For the list of genes in each GO, check Additional file 8: Spreadsheet S2.

### Gene networks

For a more holistic view of the genes influencing the eight phenotypes related to growth and meat quality we used the *p*-values and SNP additive effects (Z scored) obtained with the QXPAK.5 software to construct an AWM (see Additional file 9: Spreadsheet S3 for the list of SNP additive effects and p-values respectively used in the AWM). TCW was chosen as key phenotype for the AWM which was then used to calculate a pair-wise correlation matrix of all SNP effects. This correlation matrix was first filtered to maintain genes with correlation values equal or above 0.95. It was then used to run the PCIT methodology, which generated a gene network that consisted of 3371 genes (nodes) and 6961 gene relationships (edges) (data not shown). This is allegedly the network that contains genes more involved in growth traits because of the key phenotype (TCW), and also prone to be constituted of genes more involved with traits highly correlated to TCW, like REA and BFT. With less intensity, the network is supposed to contain genes involved with the other five phenotypes as they are less correlated to TCW. Additional file 10: Figure S6 shows a heatmap and hierarchical cluster obtained with the standardized SNP effects of the eight phenotypes that evidences that TCW, REA and BFT are more correlated than pH, CLO, LF, LM and DRE.

For visualization we used Cytoscape v3.1 and applied several visual filters to identify highly connected nodes. We suggest that the number of connections is a measure of the importance of the node to the function of the network as a whole. The assumption is that the more connections a gene has the more likely is its role regulating and influencing the function of other genes in the network. After applying the filters, the size of the node was changed to be proportional to its number of edges. The smaller the number of connections of a node, the smaller was the size of the node and vice-versa. This same rule is valid for the coloring: the more reddish, more connections and the more greenish, less connections. The obtained network shows in its center, a cluster of nodes with larger size and stronger red color intensity showing that these nodes are more interconnected and are probably more involved in key biological mechanisms underlying the phenotypes involved in the network (data not shown).

**Fig. 4:**
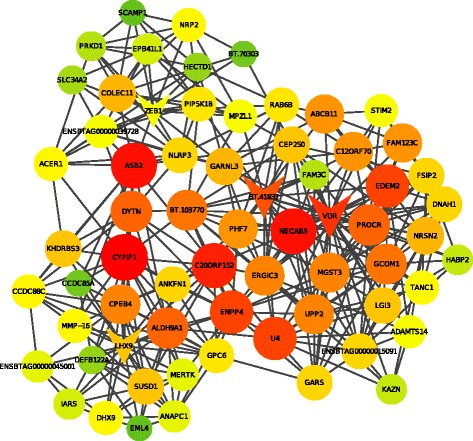
Gene network for growth and meat quality traits. Sub network for the more connected trio of transcription factors. Triangular shaped nodes show transcription factors. Greener nodes have lower connection levels than reddish nodes. Also, smaller nodes have lower connection levels than larger nodes. Labels represent gene symbols, exceptions are Ensembl IDs

As an alternative type of analysis found in similar works [35–37], we decided to divide the network in a smaller sub-network to focus on a specific set of genes that have the highest degree of connections. We used an algorithm to select the trio of transcription factors that mostly span the network with minimum redundancy [38]. Transcription factors (TF) are known to regulate several cellular mechanisms in different degrees. We arbitrarily decided to select three TF with most connections, which is a number that results in a sub-network with a reasonable sampling of the more important genes involved with the phenotypes. We used Cytoscape to create the sub-network by selecting the TF trio including the edges shared among its first degree nodes. The resulting sub-network, with 66 nodes and 359 edges, is shown in Fig. 4. Colors and node sizes represent the levels of connections inherited from the original network and triangular shaped nodes are representing the TF. The trio of TF chosen by our algorithm is composed by the VDR (Vitamin D Receptor), LHX9 (Lim Homeobox) and ZEB1 (Zinc finger E-box binding homeobox 1) genes. The VDR, or Vitamin D (1,25- Dihydroxyvitamin D3) Receptor is a hormone receptor for vitamin D3 and is a transcription factor involved in a variety of metabolic pathways. Mutations in the VDR gene can lead to the rickets disease, characterized by growth retardation, bone deformity among other effects [39] and this gene was also found related to bone density in mice [40]. More interestingly, SNPs in the coding region of VDR were found to be associated with growth traits in bovine [41]. The LHX9 gene is a transcription factor involved in developmental processes like neuronal and gonadal development [42, 43] and also to limb three-dimensional patterning and growth [44] in mice. Taken together, the known functions of LHX9 are implicated in several important growth and developmental mechanisms which suggests it is an important player in the growth trait phenotype network. The other TF from the most connected TF trio is the ZEB1 (Zinc Finger E-Box Binding Homeobox 1), a zinc finger transcription factor which is involved in cancer and in regulation of the expression of several genes (check the entry for [Uniprot:P37275] at http://www.uniprot.org), this gene have been related to muscle differentiation and myogenesis [45].

From the set of genes with direct edges to the trio of TF, there are some genes with remarkable evidence in literature relating them to growth traits, specially bone and muscle growth and adipogenesis. The ASB2 (ankyrin repeat and SOCS box containing 2), is a gene found to function in a negative regulation of muscle growth in salmon [46]. The PROCR gene (protein C receptor, endothelial) was found to positively regulate other genes positively, in an osteoblastic cell line, functioning toward bone growth [47]. The ALDH9A1 (aldehyde dehydrogenase 9 family, member A1), has a role on fetal growth in adult adipose tissue mass in bovine [48]. CPEB4, or cytoplasmic polyadenylation element binding protein 4, is associated to human waist-hip ratio [49]. The leucine-rich repeat LGI family, member 3, LGI3, is thought to have its function altered in obesity and to suppress adipogenesis [50]. GPC6 (glypican 6) is implicated in bone growth [51]. The FAM3C gene, or Family With Sequence Similarity 3, Member C was found associated to influence bone mineral density in humans [52]. A gene, the IARS (isoleucyl-tRNA synthetase), was found related to perinatal weak calf syndrome, which involves intrauterine growth retardation and low birth weights [53]. There are several other genes related to cell growth and proliferation, like ERGIC3 (ERGIC and golgi 3) [54], KHDRBS3 (KH domain containing, RNA binding, signal transduction associated 3) [55], CEP250 (centrosomal protein 250 kDa) [56], STIM2 (stromal interaction molecule 2) [57], HABP2 (hyaluronan binding protein 2) [58].

From all 66 genes present in this sub-network, 33 were found also associated to REA (32 genes) and BFT (1 gene) in the work from Tizioto et al., 2013 [7]. The rib eye area associated genes include the already discussed: VDR, ASB2, ALDH9A1, CPEB4, GPC6, FAM3C, IARS, KHDRBS3, CEP250 and STIM2. Only one gene was also found associated to BFT, EPB41L1 or Erythrocyte Membrane Protein Band 4.1-Like, which was found related to growth regulation [59]. Taken together, all the evidence found in literature of the relatedness of these genes to growth traits suggests that our methodology is reliable and the gene networks obtained shed a light on the biological mechanisms beyond growth and meat quality traits. Furthermore, as can be seen in Table 4, the SNPs that correspond to the best trio of TF have mostly *p*-values above 1e-3 meaning that this methodology captures SNPs that would not be taken as important by traditional GWAS.

**Table 4:** *P*-values of the SNPs assigned to the genes of the trio of TF (inside parentheses)

Trait	BovineHD0500009454 (VDR)	BovineHD1600024663 (LHX9)	BovineHD1300009960 (ZEB1)
TCW	2.80E-02	3.66E-02	6.66E-04
DRE	4.21E-01	4.59E-02	1.96E-01
REA	1.24E-01	2.59E-02	2.21E-03
BFT	1.00E + 00	7.42E-01	9.05E-01
LM	9.15E-01	5.01E-02	1.84E-02
LF	4.67E-01	1.60E-01	4.41E-01
PH	1.23E-01	1.00E + 00	1.00E + 00
CLO	1.27E-01	6.16E-01	5.13E-01

*TCW, Kg* Total Carcass Weight, *DRE, %* Dressing, *REA, cm* ^*2*^ Rib Eye Area, *BFT, mm* Back Fat Thickness, *LM* Lightness of Meat, *LF* Lightness of Fat, *PH* pH, *CLO* Cooking Loss

## Discussion

After the introduction of the Nelore breed in Brazil in the last century, there were some pedigree-based studies trying to characterize the founder lineages of the breed [1], but no genomic approach has been made since then. The introduction of the low and high density bovine genotyping panels, made possible the calculation and use of GRM matrices [60] in genomic similarity studies to create a genetic profile. Using only pedigree information from the 34 sires, the average inbreeding coefficient (FPED) was 3.23 % (± 3.9 SD) (calculation performed during experimental design, not shown), which is similar to the FGRM reported for sires, but lower than the FROH reported for sires. Comparing the inbreeding coefficients between sires and progeny, there is a clear decrease in inbreeding, which can only be attributed to the randomness of the genetic pool of the dams. According to recent literature, FGRM was determined as the optimal method for estimating genomic inbreeding coefficients as it can distinguish between markers that are IBS (identical by state) from markers that are IBD (identical by descendent) [61, 62]. The average FROH for either sires or progeny estimated in this study are higher than the ones reported for a Holstein population (3.8 ± 2.1 %) which have shown effects of inbreeding depression for traits in dairy cattle [62]. Also, inbreeding depression appears not to have strongly affected the phenotypes (Table 3). This result agrees with the lower inbred estimation for the progeny compared to the sires. We were not able to cross test this hypothesis by estimating inbreeding depression for the traits among sires, as we did not collect their phenotypes, altough we would expect a stronger influence of inbreeding in this case.

The average *r*^2^ values obtained in this study, considering SNPs with distance up to 150 kb apart, are higher than the values reported in the literature for Nelore and indicine breeds. Beyond 150 kb distance, the average *r*^2^ values are similar to ones found in the literature [63, 64]. However, it is worth mentioning that it is difficult to make direct comparisons among LD studies, as LD levels vary due to many factors (sample size, marker type, marker density, and population history) [65]. In this study, both maternal and paternal haplotypes were considered in contrast to studies that only consider maternal haplotypes, as it is our understanding that the Nelore population (and cattle production in general) presents higher influence from males than females and not considering paternal haplotypes would underestimate *r*^2^ values. We believe that the backcrossing with taurine local breeds, evidenced by the mtDNA origin, could have influenced the values of LD and Ne estimated for this Nelore sample.

There are some reports in the literature considering haplotype block structure in cattle, but none was found for Nelore cattle. These reports stated that with the increase in marker density, there will be more haplotype blocks of smaller sizes which will respond for higher genome coverage [66–68]. A recent study using the high density SNP panel in crossbred cattle population reports 61 % of the genome covered by haplotype blocks, and a total of 78 % of SNPs clustered into haplotype blocks [69]. The recent Ne found for Nelore in this study, is in agreement with reports of recent Ne for Holstein and Swiss Eringer breed varying from 80 to 150 individuals, and can go higher than 2000 animals for past Ne (about 2000 generations ago) [67, 70, 71]. Our results show that the diversity among sire families and lineages is not high enough to correctly separate those in a PCA, or hierarchical clustering analysis (Fig. 1). This is corroborated by the low Fst values obtained for sire families and lineages subpopulations. The uninformative values of Ne and Fst estimates were expected for the lineages as they do not represent isolated populations and should have been crosses among them.

Some of the traits used in this work were already analyzed for genome wide associations elsewhere using part of the data and with a different approach [7]. Although the phenotype data is the same in both works, our sample size was larger and the software and algorithms used for association studies were different. We have not found larger accordance than 38 %, between the set of associated genes with significance of 1e-5 to BFT, REA and LM traits, studied in that work, although we could not verify this for TCW as this trait was not present in that work. However, the quality and consistency of the genotyping data were found reliable since the PCA against other breeds showed consistent results (Fig. 1a, inset). We also checked for the fluctuation of heterozygosity along the 224,969 TagSNPs and found reasonable smoothed values of heterozygosity fluctuating around 0.3 to 0.4 (Additional file 11: Figure S7). The traits correlations were also found reasonable (see Results). Furthermore some of the top TagSNPs that had lowest p-values in the GWAS, had the corresponding gene with functional biological correlation to the main growth traits, indicating that the GWAS was able to extract some of the signal out of the noise from the data. In addition, we also performed a gene enrichment test with GOrilla, after ordering the TagSNPs for the pleiotropic effect. The pleiotropic test is a methodology to try to rank genes that are most influencing the traits in the AWM. The results showed GO terms for biological and cell adhesion and developmental growth, which are again evidence that the terms are enriched for genes related mainly to growth which is the key phenotype of the AWM. Cell adhesion is a biological process that is also found significantly enriched in the work of Tizioto et al., 2013 [7]. Cell adhesion is a process involved in the binding of a cell to a substrate which sustains its growth and therefore is intrinsically related to the growth process.

With a similar objective of the pleiotropy test, the construction of the gene networks with the AWM/PCIT [13, 35–37] is an alternative to analyze pure GWAS results as this methodology gathers the 8 GWAS results and constructs a gene network that influences the traits in a simultaneous way, but weighting accordingly to the correlations within the AWM. We decided to select TCW as the key trait for the AWM because of its reasonable heritability estimate and its congruent correlations to REA and BFT, together with the economic importance of growth traits for livestock. As the resulting network is too large (3371 nodes), we used a heuristic to divide the network into a sub-network in a way that it would be feasible to analyze and still gather most of the biological importance regarding these traits. This heuristic is implemented in an algorithm that selects the trio of TF that most span the network and its first degree nodes. As the coloring and shape of the nodes of the resulting sub-network are inherited from the original network, it can be noticed a majority of reddish and larger nodes (with more connections) indicating that the objective of the heuristic was achieved (Fig. 4). Also, a survey in the literature showed correlations between the trio of TF and several nodes from the sub-network to growth traits, suggesting that our methodology is correct. It is remarkable to note that some genes like VDR (which is the most connected TF from the network) was already associated to growth in a bovine GWAS [41] and many others have relations to the biology of growth, like proliferation and growth of muscle and bones and adipogenesis (e.g. LHX9, CPEB4, ASB2, and many others). Although comparing the results of single SNP association to the work of Tizioto et al. 2013 did not lead to more than 38 % of common associated genes, the genes from the sub-network are in 50 % of accordance with the genes associated to BFT and REA from that work. This suggests that the methodology that created this network was able to select genes that are more related to the traits involved with growth. We compared the genes from the sub-network to genes found in other studies [35–37] that also used the AWM/PCIT methodology and similar datasets (but different breeds). Although there were no great correlations, some genes in these studies were found to have similarities or to encode the same protein domains to some of the genes from the sub-network: metalloprotease and ankyrin domains (genes ADAMTS14 and ASB2 respectively), the RAB6B oncogene [36], the MER Proto-Oncogene Tyrosine Kinase (MERTK) and the SLC solute carrier gene family (SLC34A2) [35].

Taken together, these results are suggesting that the gene networks obtained are related to growth and meat quality traits and their genes should be thoroughly inspected to try to discover the biological mechanism beneath growth and meat quality phenotypes.

## Conclusion

We used high density SNP panels to genotype 34 sires, and its half-sib families, totaling 780 animals. The sires were previously selected in order to represent most of the variability of the Nelore beef cattle in Brazil. We believe this is the first work that used a genomic approach in order to try to investigate the amount of diversity of the Brazilian Nelore breed by investigating its ancestral lineages. Results showed that the population studied was not structured enough in order to differentiate families using ancestral information of sires or lineages. Eight phenotypes related to growth and meat quality were used in whole genome association studies and the results were used to construct gene networks focused on growth, using the methodology of AWM/PCIT. Literature surveys showed that both the GWAS and the gene networks constructed had significant SNPs associated to genes related to growth in former studies like the VDR, LHX9, CPEB4, ASB2 and many others. These results suggests that the methodology used to construct the gene networks can be used as an alternative approach to standard GWAS, in order to reach for novel information and to try to understand the biological mechanisms and gene compositions that leads to complex phenotypes, like growth in beef cattle.

## Methods

### Pedigree and sire lineages

Aiming to select sires that represent the bulk of genetic variability within the Nelore breed in Brazil, we consulted the main insemination centers of the country, selecting the more commercially frequent Nelore sires (polled and horned), with semen value equal or inferior to R$50.0 (around US$25.0 at the time of the study) to represent the breeders preference. Afterwards, using information from the National Zootechnical File maintained by Embrapa Beef Cattle in partnership with ABCZ (Brazilian Association of Zebu Breeders) the pedigree of all animals were assembled and a relationship pedigree matrix was created (data not shown). The sires were selected by the following rules: having the most commercialized semen among the breeders association in Brazil and ii) being the most unrelated with each other according to the value of similarity from the pedigree relationship matrix.

A list of 34 sires was assembled representing almost all lineages of the Nelore breed in Brazil (see Table 1): Karvadi, Taj Mahal, Golias, Padhu-Akasamu, Kurupathy, NO, Godar, Godhavari, Mocho GR, Nagpur, Akasamu, Padhu, Lengruber (Mundo Novo), Visual, OB, IZ and IRCA. The sire lineage was surveyed by following information about the most ancestral sire from the paternal side. Our sample study was composed of 34 sires and half-sib families with 746 animals in a total of 780 animals. The 746 calves, sons of commercial cows (not pure Nelore), were born on five different ranches, where they were raised to around 21 months old, before allocation to individual or collective pens in a feedlot located in Sao Carlos, SP, Brazil; or in Campo Grande, MS, Brazil, in an interval of 3 years. The pedigree was visualized with Cytoscape version 3.1 [72].

The mtDNA genotype was characterized as taurine (GenBank AY526085) or indicine (GenBank AY126697) from total DNA by amplification of a 366 bp fragment of mtDNA 16S (rRNA) gene using allele-specific PCR. The complete methodology can be found in [21].

### Phenotype correlations

Eight phenotypes related to growth and meat quality were collected: Total Carcass Weight (TCW, Kg); Dressing percentage (DRE, %); Rib Eye Area (REA, cm2); Back Fat Thickness (BFT, mm); Lightness of Meat (LM); Lightness of Fat (LF); pH (PH); Cooking Loss (CLO, %). The phenotypes were collected as described elsewhere [7], see Table 2. The number of animals with phenotype measurements varied around 670. The VCE software (ftp://ftp.tzv.fal.de/pub/vce6/) was used to calculate the correlations between phenotypes, the heritabilities and the respective standard errors (SE). The model used was the following (Equation 1):

1 $$ \mathrm{y} = \mathrm{X}\upbeta + \mathrm{Zg} + \mathrm{E} $$

Where y is a vector with the values for a given phenotype, X and Z are incidence matrix, β is a vector for fixed effects composed of the first principal component used as co-variate, Slaughter Age also a co-variate, the contemporary group effect is represented by Origin * Year of Birth and Animal ID, representing the pedigree. g is a vector of additive genetic effects, normally distributed g ~ N(0, *σ*_*g*_^2^). E represent the vector of residual effects, E ~ N(0, *σ*_*e*_^2^).

### Genotyping and quality control

All 34 sires and 746 half-sibs were genotyped with a high density SNP panel (Illumina Bovine HD SNP Chip). The DNA sample collection and the SNP chip genotyping were performed as previously described [7]. We used quality control filters for minor allele frequency (5 %) and call rate for sample and SNPs (95 %). Filters were applied using PLINK [73] and Biocoductor/R [74, 75]. This filters yielded a dataset comprised of 449,203 SNPs which was used for linkage disequilibrium and haplotype block description using BEAGLE [76] for genotype phasing and missing genotype imputation and LDexplorer [77] for haplotype block recognition following Gabriel et al. 2002 [78] algorithm, and for estimating effective population size (Ne) according to [79].

### Genomic relationship matrix

The genomic relationship matrix (GRM) was constructed [60, 80] using the TagSNPs. The GRM was used to estimate genomic inbreeding coefficient (FGRM), estimated as the diagonal elements of the GRM [60], and to perform PCA analyzes with all 780 Nelore samples and the same TagSNPs for 46 samples of the Brahman breed, 36 samples of Hereford breed and 44 samples of Angus breed, genotyped with the same high density SNP panel from the Hapmap project [18].

### Genome Wide Association Study (GWAS)

All GWAS were performed using the software QXPAX5 [28]. Although we have used the first principal component of the PCA analysis as covariate to correct for population substructure bias [81], we needed to perform a manual correction to fix for contemporary group substructure bias, as follows. We calculated the mean phenotype value for each contemporary group and the raw phenotype value of every animal from a given group was subtracted from its correspondent mean contemporary group value. The model used was the one that follows (Equation 2), now without the contemporary group covariate:

2 $$ y{\hbox{'}}_{ij}=X{\beta}^{\hbox{'}} + Zg+{S}_k{s}_{jk} + {E}_{ij} $$

Where *y* ' is a vector containing the phenotype corrected for the contemporary group effect from the i-th animal to the j-th trait (check Table 2), *X* is the incidence matrix relating fixed effects in *β*' with observations in *y*'_*ij*_. *β*' is a vector for fixed effects composed of the first principal component used as co-variate, slaughter age also a co-variate, representing the pedigree. *Z* is the incidence matrix relating random additive polygenic effects in *g* with the observations in *y*'_*ij*_. *g* is a vector composed of the additive genetic effects, normally distributed *g* ~ *N*(0, *Aσ*_*g*_^2^); were *A* represents the numerator relationship matrix derived from the pedigree. *S*_*k*_ is the vector of genotypes for the k-th SNP across all animals. *s*_*jk*_ is a vector that represents the additive effect of the k-th SNP on the j-th trait and *E* represents the vector of residual effects, *E* ~ *N*(0, *σ*_*e*_^2^). QXPAK5 was run for the eight phenotypes using the TagSNP set. The p-values and additive genetic values for each SNP were obtained for each phenotype and used to construct the AWM matrix. The FDR (false discovery rate) was calculated using the formula of [33].

### AWM/PCIT and gene networks

AWM is a methodology to explore the results obtained from several GWAS (many traits) and generate a matrix of genes (or SNPs) co-associated in a pair-wise manner to these traits. The rows in the AWM are composed of the genes (or SNPs) and the columns represent the traits. The AWM matrix is filled with the additive effects of the SNPs. AWM was used to select SNPs associated to the key trait (TCW) and also SNPs associated to several (three or more) other traits. This was done in order to keep SNPs that are important to “growth” in a holistic manner. Afterwards, a pair-wise Pearson’s correlation is calculated and the data is submitted to PCIT that is a network inference methodology used to construct the gene network. The AWM was constructed as described elsewhere [82]. The correlation matrix was calculated for every entry of the AWM, using the standardized Z score (the additive SNP effect divided by the standard deviation) within the R environment. The correlation matrix was used as input for the PCIT package for R [16]. A list of genes that correspond to each TagSNPs were obtained from Ensembl version 74 (http://www.ensembl.org), where genes were assigned to its nearest TagSNP. Gene networks generated by PCIT were visualized with Cytoscape version 3.1 [72]. A transcription factor list was obtained from the Animal Transcription Factor Database [83] and the most connected trio of transcription factors from the gene network was obtained as described elsewhere [38].

### Pleiotropy and gene enrichment

To test for pleiotropy we employed the approach described by [33]. In brief, the effects of the 224,969 TagSNPs estimated from the GWAS were divided by their associated standard errors to obtain a t-value corresponding to the studentized SNP effects. A multi-trait test of the i-th TagSNP was performed by storing its studentized effects across the 8 traits in the 8 x 1 vector *t*^*i*^. Then, the quadratic form *t*_*i*_'*V*^− 1^*t*_*i*_, where *V* is the correlation matrix among the SNP effects, is distributed approximately as a chi-squared with 8 df under the null hypothesis that the TagSNP does not affect any of the traits [33]. The TagSNPs where then ordered according to the results and the ordered list of corresponding genes were used as input to GOrilla [34].

### Linkage disequilibrium and other analyses

The relationship between LD (*r*^2^) and Ne can be expressed by the Equation 3:

3 $$ {r}^2=1\ /\ \left(4cNe+1\right) $$

where, *c* is the genetic distance between two SNP expressed in Morgans [84]. Considering each SNP pairs located within 100Mbp of the same autosomal chromosomes, with physical distances between SNP converted to genetic distances by the simple assumption of 1 cM ~ 1 Mb were used for Ne estimation. Haploview was used for obtaining TagSNPs, which were used for deriving inbreeding coefficient by runs of homozygozity (FROH), calculated with PLINK, as described by [62]. Heterozygosity changes along the 224,969 TagSNPs were calculated with PLINK and plotted in R using a smoothing function from the lokern package, one point for each 100 TagSNPs, as described elsewhere [80]. The Wright’s F statistics (Fst) analyses were run with PLINK 1.9 (https://www.cog-genomics.org/plink2) using the TagSNPs of the 780 animals, separated: i) by lineage (17 subpopulations) and ii) by sire families (34 subpopulations).

### Ethics approval

All experimental procedures involving steers were approved by the Institutional Animal Care and Use Committee Guidelines from Brazilian Agricultural Research Corporation – EMBRAPA and sanctioned by the president of the committee, Dr. Rui Machado.

## Availability of supporting data

The data set supporting the results of this article is included within the article (and its additional files). Genotypic data is available upon request depending on a signed declaration of exclusive research purpose.

## Additional files

Additional file 1: Figure S1. Genetic profiling using the Genomic Relationship Matrix. (A). PCA analysis of all 780 Nelore (lineages differentiated by colors). (B). Heatmap and hierarchical clustering of the 780 Nelore. Lateral palette colors represent the families; upper color palette represent the lineages (same color correspondence to A); shades of grey from the heatmap represent relationship similarities (darker is less related). (C). Pedigree view of the families showing the sires (blue), sibs (green), dams (pink) and the lineage ancestral from father side (red). (PDF 1027 kb) Additional file 2: Figure S2. Overall autosomes average *r* ^2^ values for Nelore animals with respect to physical genomic distance (kb) and its confidence interval (0.05). (PDF 5 kb) Additional file 3: Table S1. Haplotype block summary per autosome (BTA). (XLSX 6 kb) Additional file 4: Figure S3. Haplotype block distributions for autosome chromosomes (BTA) in Nelore cattle. (PDF 296 kb) Additional file 5: Figure S4. Decay of Ne over ten generations. (PDF 82 kb) Additional file 6: Figure S5. QQ-plots from the GWAS results. (A-H) panels show the QQ-plots for: A. Total Carcass Weight (TCW); B. Dressing % (DRE); C. Rib Eye Area (REA); D. Back Fat Thickness (BFT); E. Lightness of Meat (LM); F. Lightness of Fat (LF); G. pH (PH); H. Cooking Loss (CLO). (PDF 302 kb) Additional file 7: Spreadsheet S1. List of genes tagged by significantly associated SNP in common to this report and Tizioto et al. 2013. (XLSX 16 kb) Additional file 8: Spreadsheet S2. Genes found enriched according to GOrilla. Genes present in the GO:0007155 (“cell adhesion”); GO:0022610 (“biological adhesion”) and GO:0048589 (“developmental growth”), after running GOrilla using an ordered list of genes after a pleiotropy test. (XLSX 14 kb) Additional file 9: Spreadsheet S3. AWM populated with SNP additive effects and p-values (4047 entries). Also genes assigned to each SNP in symbol and ENSEMBL format. (XLSX 747 kb) Additional file 10: Figure S6. Heatmap and hierarchical plot of the AWM/PCIT matrix. Hierarchical plot using the AWM/PCIT correlation matrix (standardized values) for the eight traits. Total Carcass Weight (TCW); Dressing % (DRE); Rib Eye Area (REA); Back Fat Thickness (BFT); Lightness of Meat (LM); Lightness of Fat (LF); pH (PH); Cooking Loss (CLO). (PDF 113 kb) Additional file 11: Figure S7. The fluctuation of heterozygosity along the 224,969 tagSNPs. Red line shows smoothed dataset (one point every 100 tagSNPs). (PDF 269 kb)

## Abbreviations

ABCZ: Brazilian Association of Zebu Breeders
AWM: Association Weight Matrices
BFT: back fat thickness
CLO: cooking loss
DRE: dressing
FDR: false discovery rate
FGRM: average genomic inbreeding coefficient from the GRM
FPED: average inbreeding coefficient derived from pedigree
FROH: inbreeding coefficient derived from runs of homozygosity
Fst: Wright’s f statistic
GO: gene ontology
GRM: genomic relationship matrix
GWAS: genome wide association studies
IBD: identical by descendent
IBS: identical by state
LD: linkage disequilibrium
mtDNA: mitochondrial DNA
Ne: effective population size
PCA: principal component analysis
PCIT: partial correlation and information theory
PH: pH
*r*^2^: squared correlation coefficient
REA: rib eye area
SD: standard deviation
SE: standard error
SNP: single nucleotide polymorphism
TCW: total carcass weight
TF: transcription factors
