# Erratum to: ‘Genomic structure and marker-derived gene networks for growth and meat quality traits of Brazilian Nelore beef cattle’

**DOI:** 10.1186/s12864-016-2856-2

**Published:** 2016-07-13

**Authors:** Maurício A. Mudadu, Laercio R. Porto-Neto, Fabiana B. Mokry, Polyana C. Tizioto, Priscila S. N. Oliveira, Rymer R. Tullio, Renata T. Nassu, Simone C. M. Niciura, Patrícia Tholon, Maurício M. Alencar, Roberto H. Higa, Antônio N. Rosa, Gélson L. D. Feijó, André L. J. Ferraz, Luiz O. C. Silva, Sérgio R. Medeiros, Dante P. Lanna, Michele L. Nascimento, Amália S. Chaves, Andrea R. D. L. Souza, Irineu U. Packer, Roberto A. A. Torres, Fabiane Siqueira, Gerson B. Mourão, Luiz L. Coutinho, Antonio Reverter, Luciana C. A. Regitano

**Affiliations:** Embrapa Agricultural Informatics, Av. André Tosello, 209, Campinas, SP Brazil; Embrapa Southeast Livestock, Rodovia Washington Luiz, Km 234, São Carlos, SP Brazil; Commonwealth Scientific and Industrial Research, Organization - Agriculture, 306 Carmody Road, Brisbane, QLD Australia; Department of Genetics and Evolution, Federal University of São Carlos, Rodovia Washington Luiz, Km 235, São Carlos, SP Brazil; Embrapa Beef Cattle, Av. Rádio Maia, 830, Campo Grande, MS Brazil; State University of Mato Grosso do Sul, Rodovia Uems-Aquidauana km 12, Aquidauana, MS Brazil; Department of Animal Science, University of São Paulo, Av. Padua Dias, Piracicaba, SP 11306 Brazil; Faculdade de Medicina Veterinaria e Zootecnia, Federal University of Mato Grosso do Sul, Av. Senador Filinto Müller, Campo Grande, MS 2443 Brazil

## Erratum

Unfortunately, the original version of this article [1] contained an error. Table 2 values were included incorrectly. The correct values can be found below. These values should also be indicated under the “Data inspection” subsection, first paragraph found under “Gene networks for growth and meat quality traits” section:

**Table 2:** Trait data and GWAS results information

Trait	n	min	max	avr	SD	h^2^	p ≤ 10 − 3	p ≤ 10 − 4	p ≤ 10 − 5
TCW	671	182.5	346.8	250.26	27.88	**0.254**	1246	219	32
						**(0.042)**	(17.97)	(10.26)	(7.03)
DRE	671	42.6	86.5	56.22	3.63	**0.096**	680	100	20
						**(0.031)**	(33.02)	(22.49)	(11.25)
REA	669	39.24	84.43	60.45	7.26	**0.473**	826	157	31
						**(0.049)**	(27.16)	(14.32)	(7.26)
BFT	669	0.07	20	6.16	2.25	**0.154**	1723	305	56
						**(0.041)**	(12.97)	(7.37)	(4.02)
LM	671	33.5	49.43	40.09	3.18	**0.045**	957	218	64
						**(0.030)**	(23.43)	(10.31)	(3.51)
LF	**671**	**16.54**	**84.36**	**75.65**	**4.45**	**0.049**	1388	340	96
						**(0.021)**	(16.12)	(6.61)	(2.34)
PH	**666**	**5.00**	**6.9**	**5.54**	**0.21**	**0.026**	928	215	56
						**(0.022)**	(24.17)	(10.45)	(4.02)
CLO	**667**	**14.42**	**66.90**	**28.71**	**5.21**	**0.099**	939	138	22
						**(0.041)**	(23.88)	(16.29)	(10.22)

“…higher estimates were observed for rib eye area (REA; h^2^ = **0.473**), total carcass weight (TCW; h^2^ = **0.254**) and back fat thickness (BFT; h^2^ = **0.154**). There was a negative correlation between BFT and REA (-**0.604**) and between BFT and TCW (-**0.632**) and a strong positive correlation between TCW and REA (**0.762**) which makes biological sense indicating that the data is consistent.”
