# Supplementary figures and images for: Genomic structure and marker-derived gene networks for growth and meat quality traits of Brazilian Nelore beef cattle
Source: BMC Genomics. 2016 Mar 15;17:235. doi: 10.1186/s12864-016-2535-3 (PMC4791965; doi:10.1186/s12864-016-2535-3)

**A**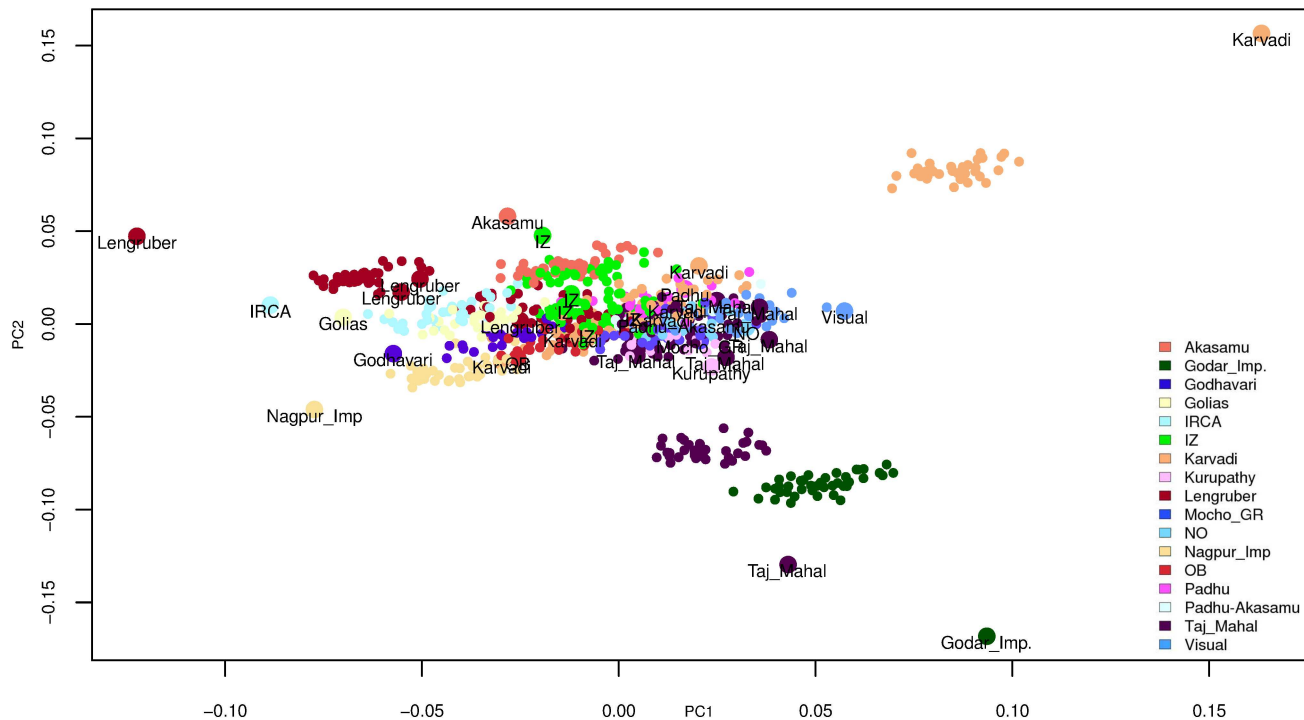**B**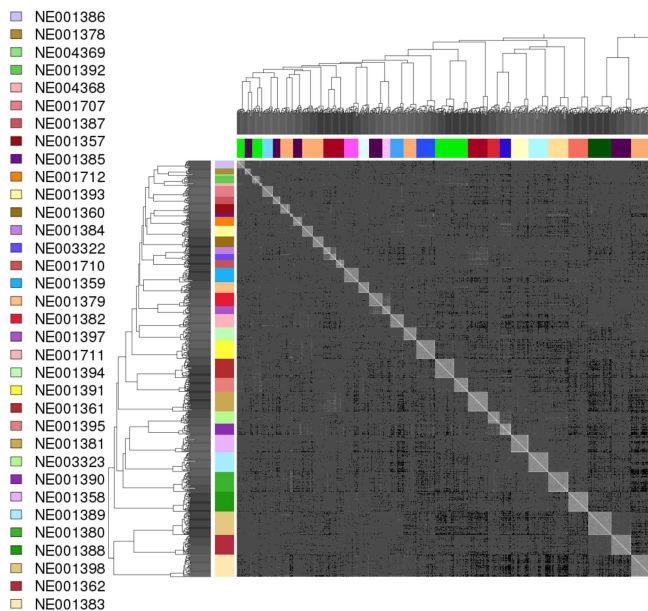**C**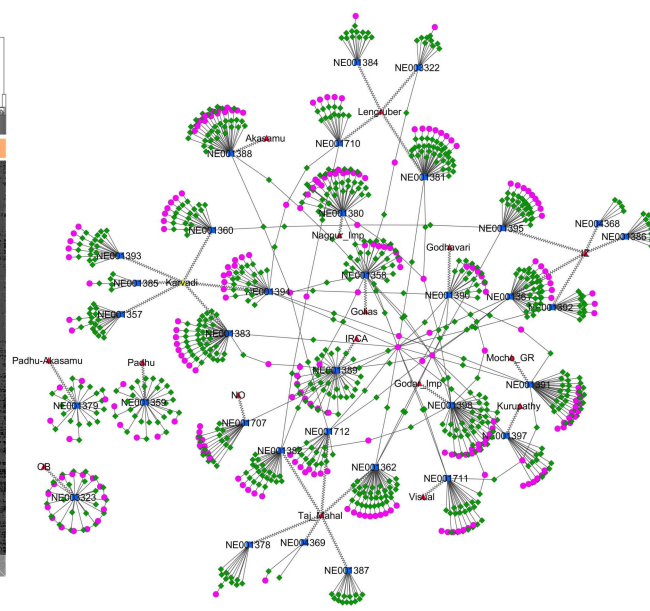

Supplement: Additional file 1: — Figure S1. Genetic profiling using the Genomic Relationship Matrix. (A). PCA analysis of all 780 Nelore (lineages differentiated by colors). (B). Heatmap and hierarchical clustering of the 780 Nelore. Lateral palette colors represent the families; upper color palette represent the lineages (same color correspondence to A); shades of grey from the heatmap represent relationship similarities (darker is less related). (C). Pedigree view of the families showing the sires (blue), sibs (green), dams (pink) and the lineage ancestral from father side (red). (PDF 1027 kb) [file 12864_2016_2535_MOESM1_ESM.pdf]

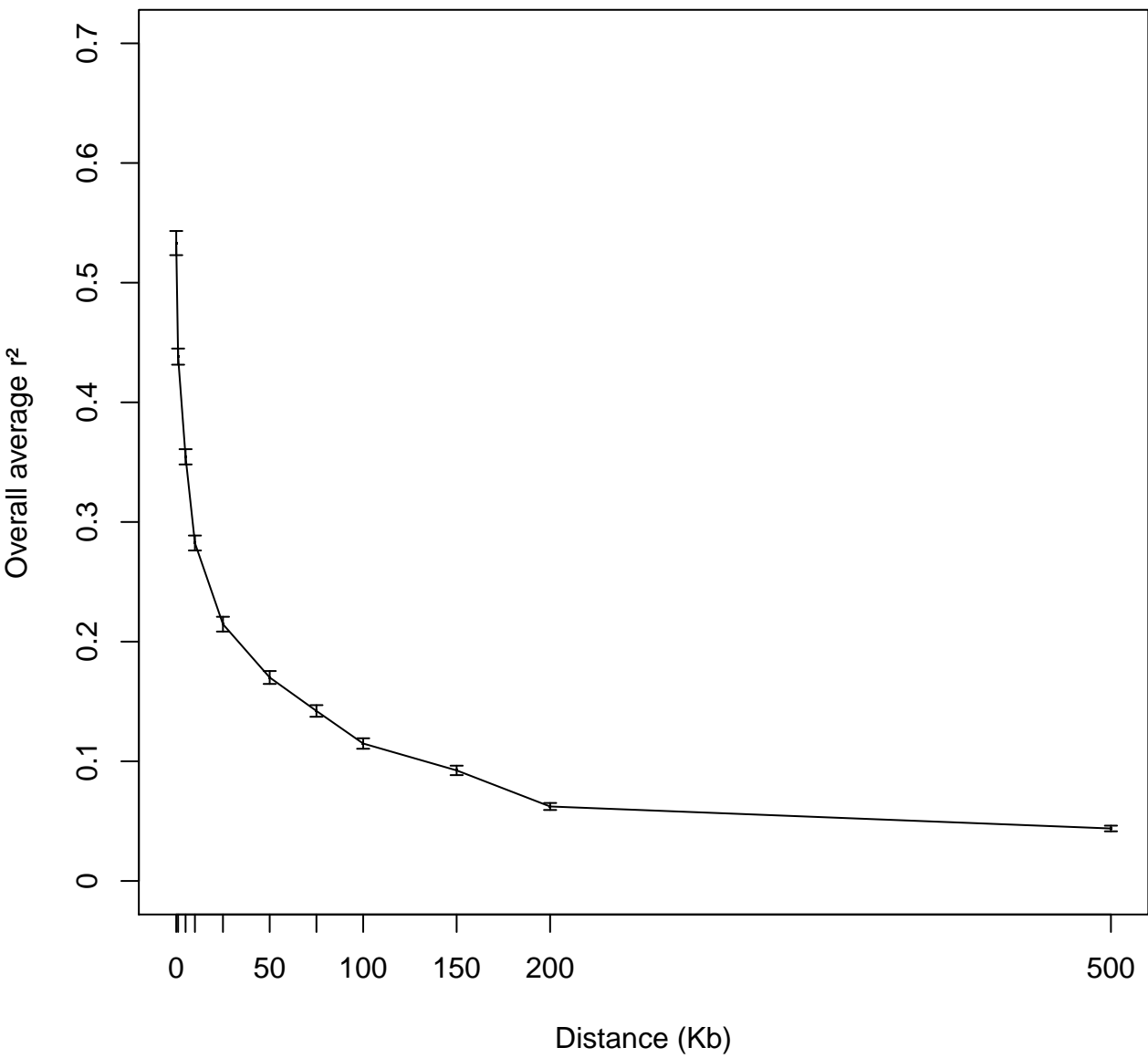

Supplement: Additional file 2: — Figure S2. Overall autosomes average r 2 values for Nelore animals with respect to physical genomic distance (kb) and its confidence interval (0.05). (PDF 5 kb) [file 12864_2016_2535_MOESM2_ESM.pdf]

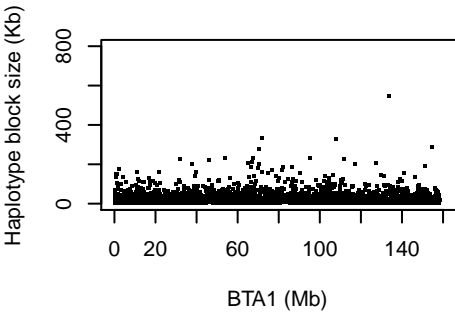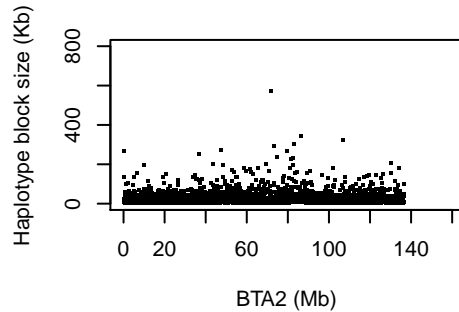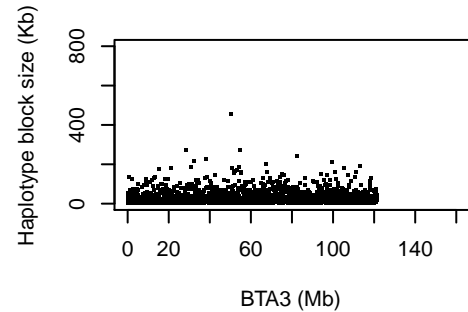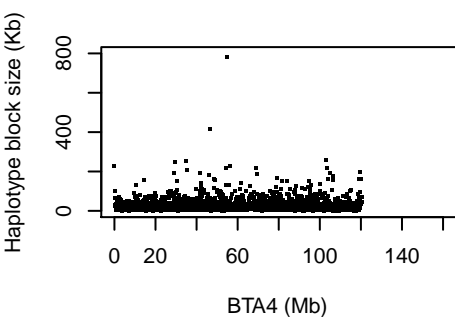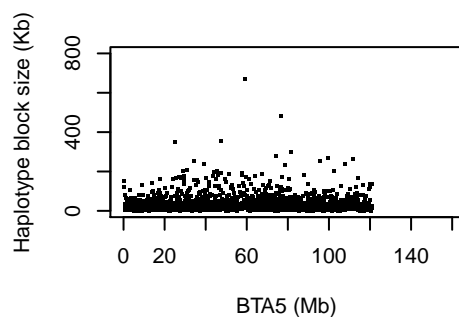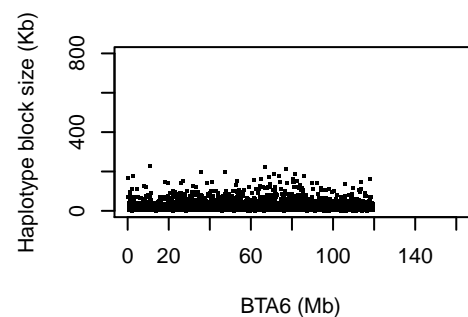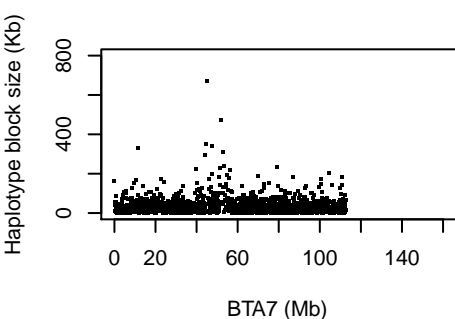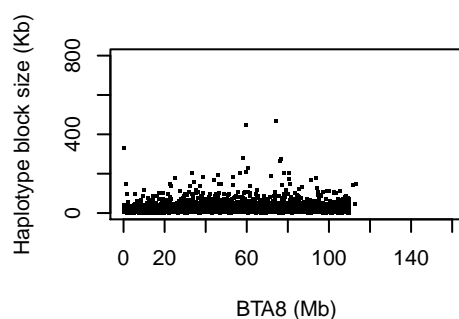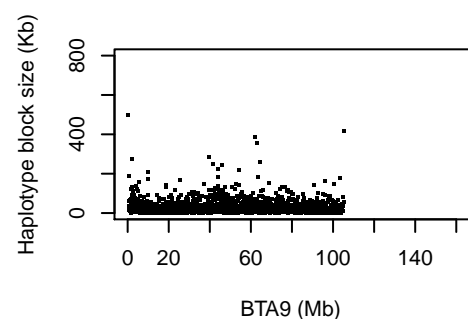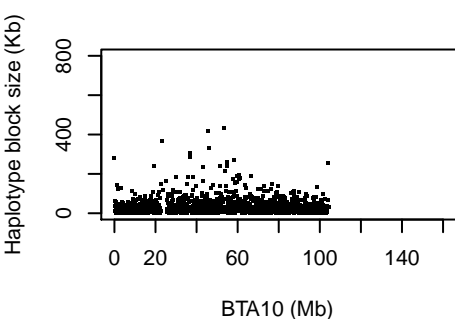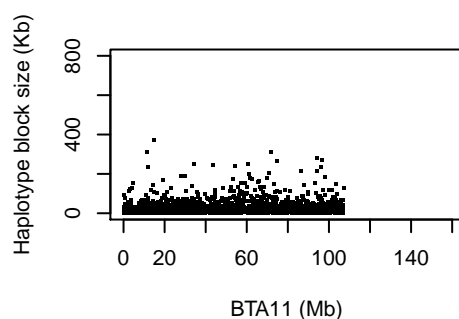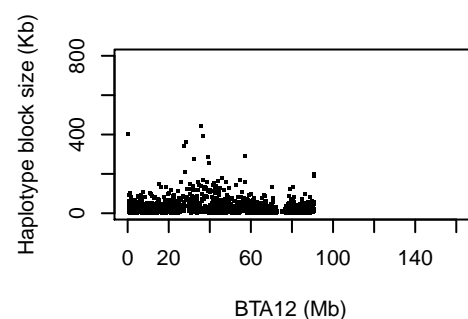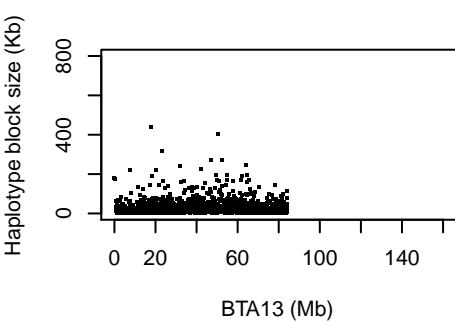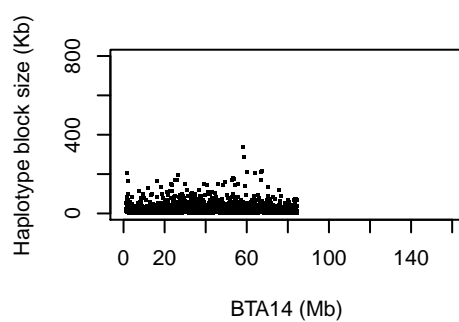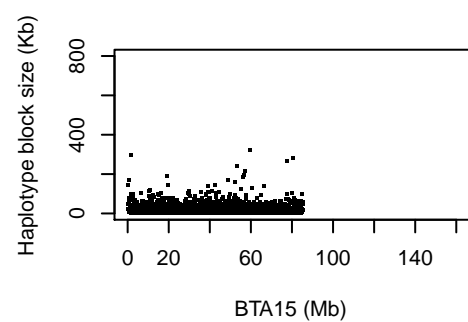

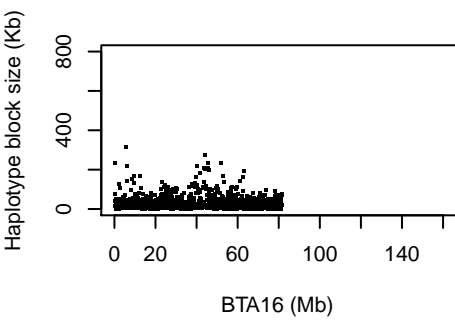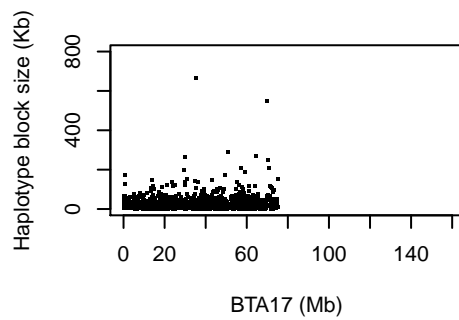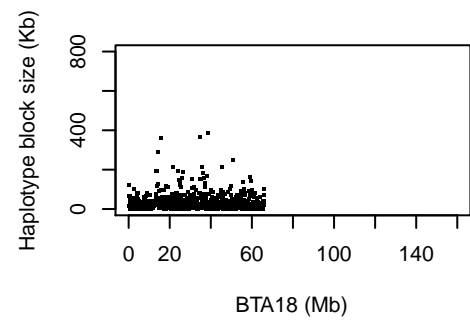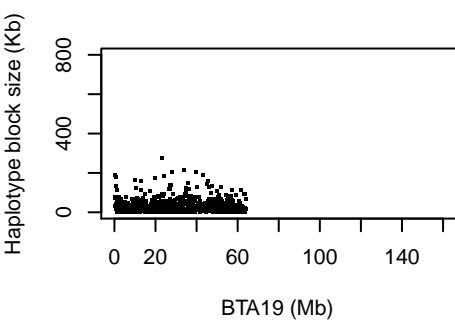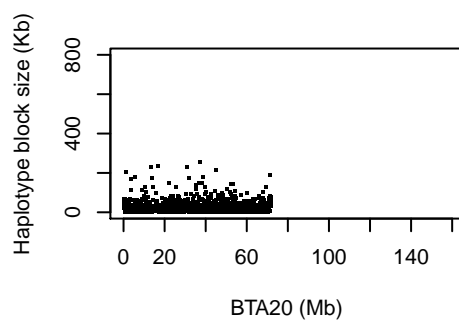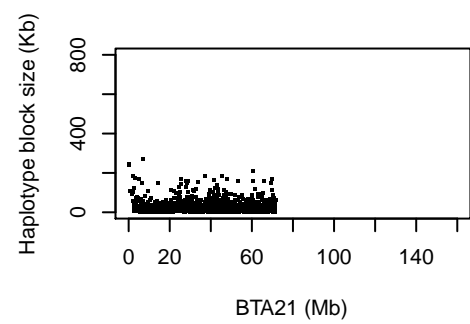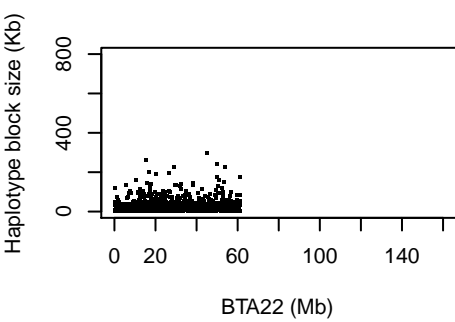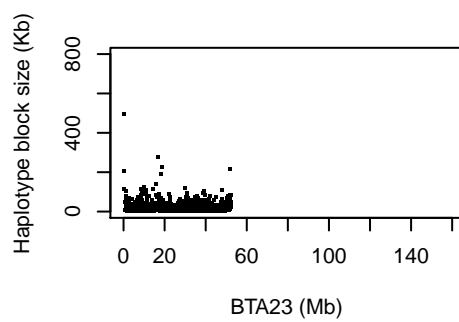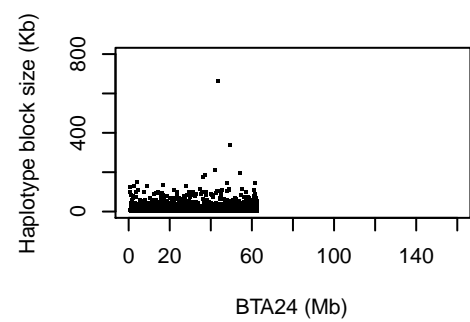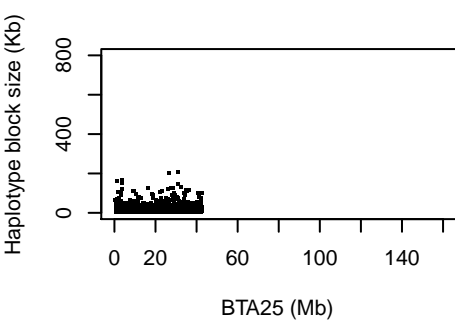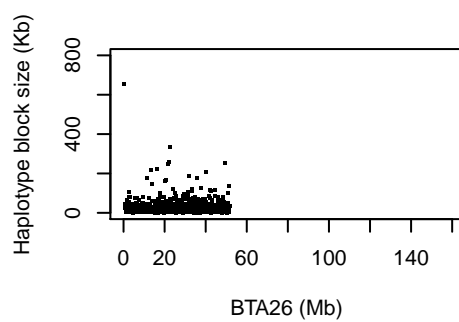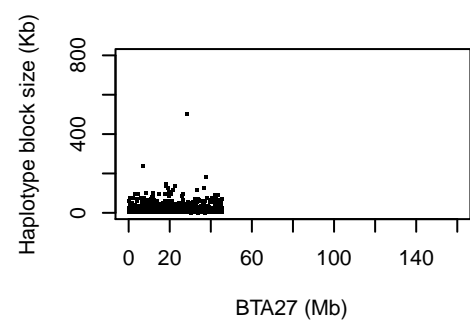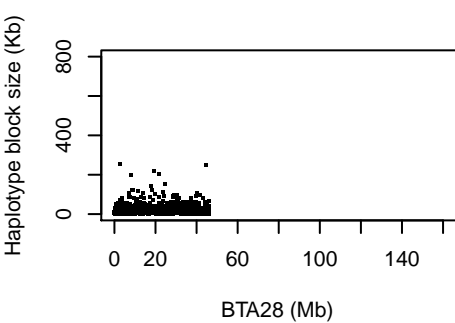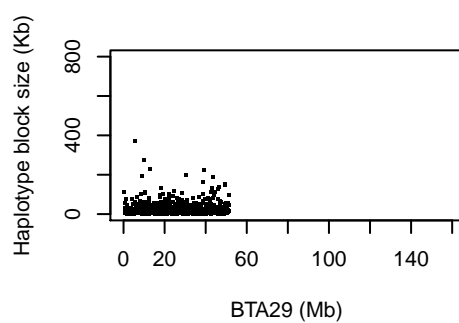

Supplement: Additional file 4: — Figure S3. Haplotype block distributions for autosome chromosomes (BTA) in Nelore cattle. (PDF 296 kb) [file 12864_2016_2535_MOESM4_ESM.pdf]

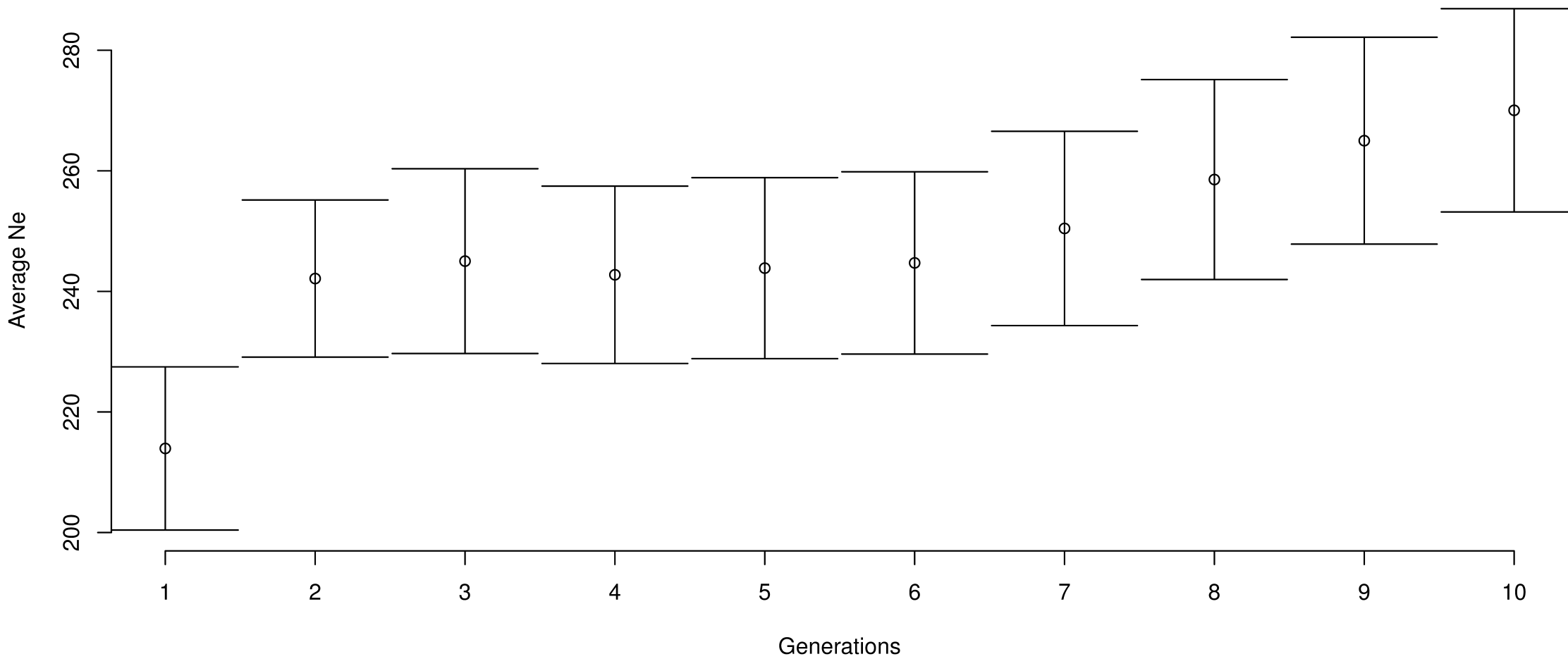

Supplement: Additional file 5: — Figure S4. Decay of Ne over ten generations. (PDF 82 kb) [file 12864_2016_2535_MOESM5_ESM.pdf]

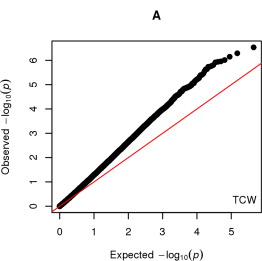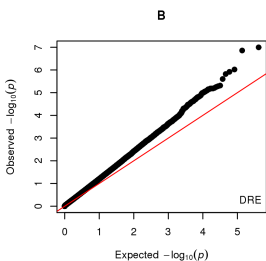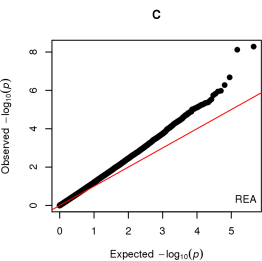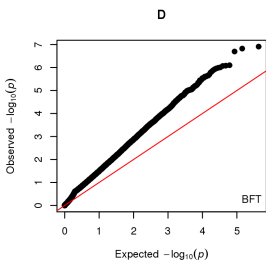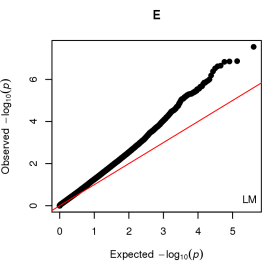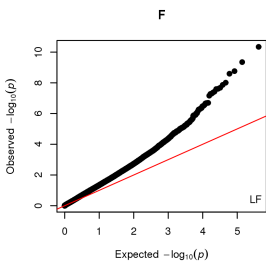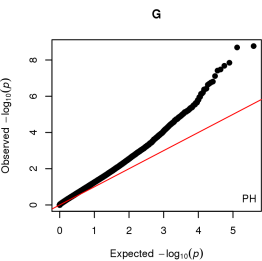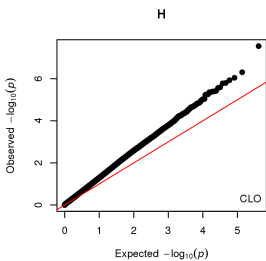

Supplement: Additional file 6: — Figure S5. QQ-plots from the GWAS results. (A-H) panels show the QQ-plots for: A. Total Carcass Weight (TCW); B. Dressing % (DRE); C. Rib Eye Area (REA); D. Back Fat Thickness (BFT); E. Lightness of Meat (LM); F. Lightness of Fat (LF); G. pH (PH); H. Cooking Loss (CLO). (PDF 302 kb) [file 12864_2016_2535_MOESM6_ESM.pdf]

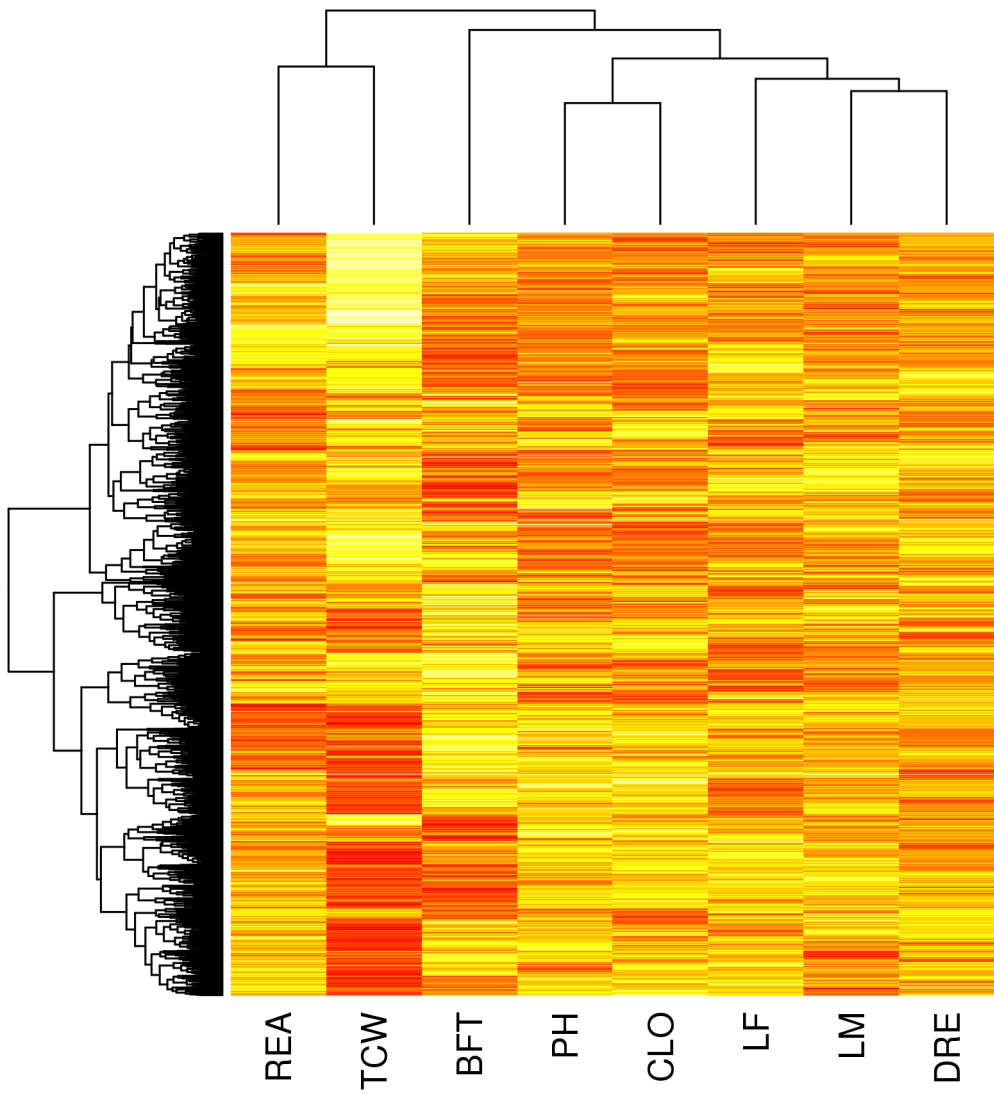

Supplement: Additional file 10: — Figure S6. Heatmap and hierarchical plot of the AWM/PCIT matrix. Hierarchical plot using the AWM/PCIT correlation matrix (standardized values) for the eight traits. Total Carcass Weight (TCW); Dressing % (DRE); Rib Eye Area (REA); Back Fat Thickness (BFT); Lightness of Meat (LM); Lightness of Fat (LF); pH (PH); Cooking Loss (CLO). (PDF 113 kb) [file 12864_2016_2535_MOESM10_ESM.pdf]

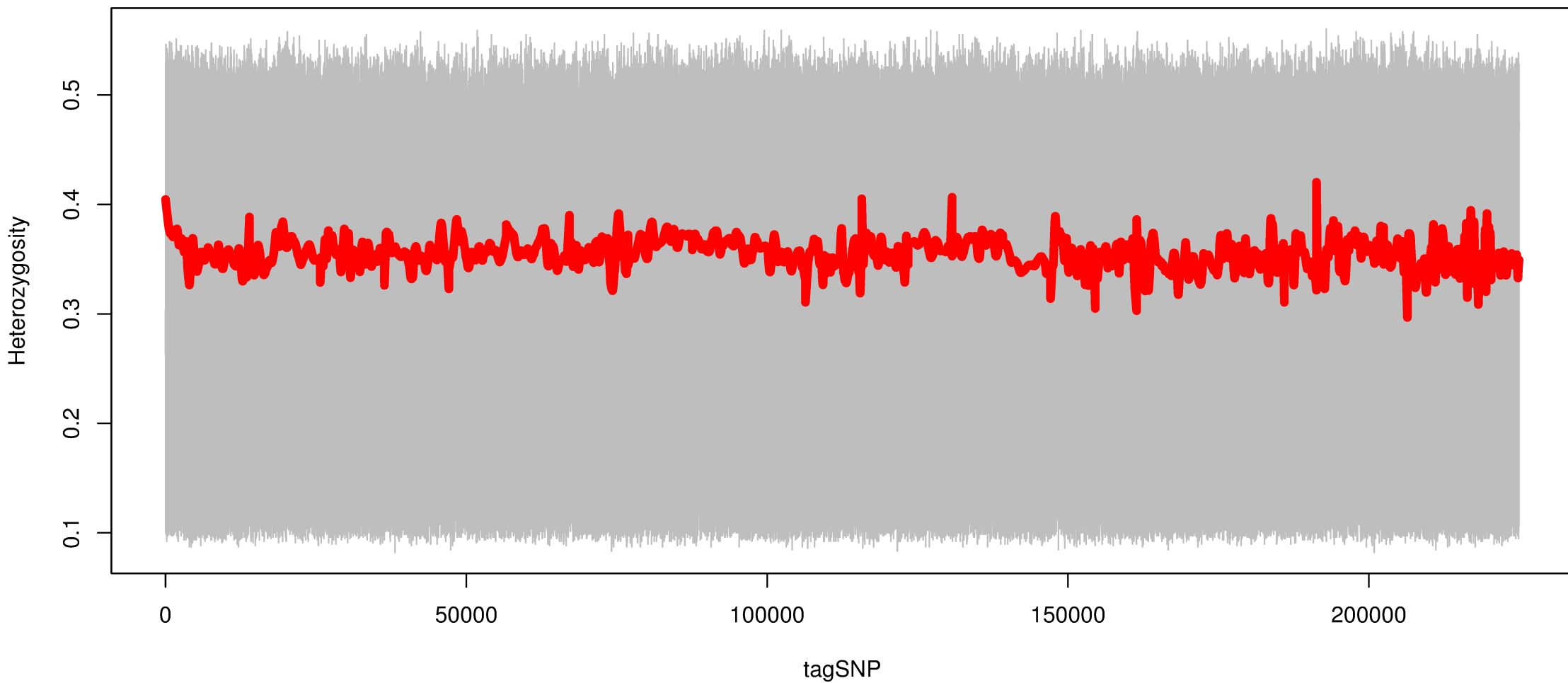

Supplement: Additional file 11: — Figure S7. The fluctuation of heterozygosity along the 224,969 tagSNPs. Red line shows smoothed dataset (one point every 100 tagSNPs). (PDF 269 kb) [file 12864_2016_2535_MOESM11_ESM.pdf]
